# Analysis of Tool Wear and Roughness of Graphite Surfaces Machined Using MCD and NCD-Coated Ball Endmills

**DOI:** 10.3390/mi13050766

**Published:** 2022-05-13

**Authors:** Hyeonhwa Lee, Jinsoo Kim, Jeongyeon Park, Jongsu Kim

**Affiliations:** Molding & Metal Forming R&D Department, Korea Institute of Industrial Technology, Bucheon 14441, Korea; vikar02@kitech.re.kr (H.L.); kjs75111@kitech.re.kr (J.K.); parkjy@kitech.re.kr (J.P.)

**Keywords:** diamond coating, delamination wear, abrasion, coating adhesion, crystal/grain size, surface roughness, rough machining, precision machining

## Abstract

The high-purity G5 graphite material is widely used for glass moulding and provides high hardness and brittleness because it is sintered to fine particles unlike other graphite materials. Hence, tool cutting of a G5 workpiece is performed by local fracture instead of plastic deformation of the machined surface. Although a diamond-coated tool with outstanding hardness is used to machine very hard graphite, the tool shows variability regarding the service life and machining performance depending on the grain size, even in the same machining environment. We investigated the wear and change trend of machined surface roughness considering microcrystalline diamond (MCD) and nanocrystalline diamond (NCD)-coated tools, which are generally used to machine graphite materials, and analysed their relation with coating. For rough machining, the MCD-coated tool, for which the delamination of coating occurred later, showed less wear and improved machined surface roughness. For precision machining, the NCD tool showed less tool wear rate relative to the cutting length, leading to a small difference in the machined surface roughness between the two tools. We conclude that if rough and precision machining processes are performed using the same cutting tool, the MCD-coated tool is advantageous in terms of service life, while the difference in roughness of the final machined surface between the tools is negligible.

## 1. Introduction

The high-purity G5 graphite material is widely used for glass moulding or machine parts that require thermochemical stability. Unlike general graphite, G5 is composed of micro-grains and has excellent mechanical properties, such as high hardness and strength. According to Ashby [1], owing to its excellent chemical stability, G5 is suitable for thermocompression moulding because it has fewer chemical effects on both glass and graphite moulds. Moreover, given the similar coefficients of thermal expansion of G5 and glass, G5 can provide high-dimensional and shape accuracies when forming glass [2], being also advantageous for machine parts in silicon ingot production. However, as G5 exhibits brittleness and hardness, the workpiece surface may be easily broken during mechanical machining, and the blade of the cutting tool may be easily damaged. Wang et al. [3] and Cabral et al. [4] analysed the machining mechanism of graphite materials, the shape and size of fragments, and their effects on the cutting tool from various angles. Cabral et al. [4] claimed that a diamond-coated tool, which is highly resistant to abrasion, is suitable for graphite machining because local fracturing causes grinding of the machined surface and produces fine graphite particles in the form of powder, consequently grinding the cutting tool. Wang et al. [3] explained that high-speed precision cutting of high-purity graphite is essential for the performance and duration of a mould and reported that graphite fragments with irregular shapes cause severe friction and impact on the milling tool and workpiece. Therefore, they suggested that diamond-coated tools are suitable for high-speed precision machining of high-purity graphite moulds and reported comparative experimental results by applying various types of diamond coatings [3,5].

Previous studies have shown that the wear resistance of cutting tools is a critical factor for high-precision machining of high-purity graphite materials. Moreover, while graphite grains with high hardness wear the tool surface, the milling process affects the delamination of the coating layer through repetitive impacts with the workpiece caused by intermittent cutting. In general, the life of a coating film drastically drops after the film delaminates from the substrate [6]. Therefore, it is necessary to carefully track and analyse the delamination times of various diamond-coating films as well as the wear of tools and change of machined surface roughness throughout high-speed milling and cutting. Furthermore, the endmill type notably influences tool wear and machinability [5]. Thus, the diamond-coated endmill type should also be investigated. Wang et al. [5] claimed that using a corner radius endmill is better than using a flat endmill for high-purity graphite machining because tool tipping can occur easily when using the flat endmill owing to the nature of the material.

Various studies have been conducted on the service life and machinability of tools according to the diamond grain size, deposition method, cutting parameters, and cutting-edge shape of diamond-coated cutting tools [7,8,9,10,11]. Bian et al. [7] closely analysed two coated tools in ductile mode machining of materials with high hardness and brittleness through a micro-milling experiment on ZrO_2_ ceramic materials of normal crystalline and nanocrystalline-coated flat endmills. They could perform mirror surface precision machining with a surface roughness average Ra of 0.02 μm for the two coated tools under suitable conditions and Ra of 0.1 μm or lower regardless of the cutting condition for the nanocrystalline-coated tool. However, the delamination of coating can occur faster with nanocrystalline coating. Suwa et al. [9] investigated the cutting performance of a diamond-coated ball endmill for milling a sintered tungsten carbide with varying cobalt content. They also analysed the effect of the morphology of the cutting edge and found that a cutting edge with an S shape showed a better cutting performance than that with a straight shape. The evaluation and analysis of the ear and machining characteristics of diamond-coated endmill tools according to diamond coating, workpiece, and endmill types are essential and constitute a current research topic.

We experimentally analysed the coating wear and delamination time of ball endmills according to the diamond coating type during high-speed milling of a very hard graphite moulding material. We also analysed the change of the machined surface roughness owing to milling. The presentation of the wear and machined surface characteristic data of two diamond ball endmills for graphite moulds that require high-precision surface quality will allow the comparison between existing flat and corner radius ball endmill tools. In addition, the data may provide guidance on the selection of appropriate tools for high-speed precision machining of high-purity graphite.

## 2. Experimental Setup

### 2.1. Preparation of Workpiece and Diamond-Coated Tool

#### 2.1.1. High-Purity Graphite Workpiece

Graphite is a polycrystalline material with six carbon rings connected in a hexagonal planar lattice to form an overlapping layer. Graphite is more susceptible to local fracture than to plastic deformation during high-speed machining because it has a higher brittleness than metal. According to Wang et al. [3], the effect of graphite on the tool cutting edge varies according to the direction of the base surface of the crystal structure. The graphite workpiece used in this study was a commercial graphite grade glass 5 (G5; MERSEN, Newburyport, MA, USA) sample, which is often used in the glass moulding press owing to its high hardness and strength compared with general graphite [5]. A general product sintered with a grain size of around 7 μm was selected with a bulk density of 1.84 g/cm^3^ for this study. The mechanical properties of the G5 workpiece are summarised in Table 1.

#### 2.1.2. Diamond-Coated Endmill Tool

Commercial tools were selected for cutting (milling). The tools were prepared with two types of diamond coatings with the same geometry. The selected commercial tool was an S-shape ball endmill with two blades (2DRB-020-160-660; HAN SONG, Hwaseong-City, South Korea). The cutting-edge shape of the ball endmill influences machinability. Suwa et al. [9] confirmed that an S shape has better machinability than a straight shape for graphite machining. Accordingly, the S shape was selected for the ball endmill cutting edge in this study. The tool specifications are listed in Table 2.

The substrate of the ball endmill was made of a tungsten carbide–cobalt (WC-Co) alloy containing 6% of cobalt and 1 μm or smaller tungsten carbide micro-grains (see Table 3). A finer grain reduces the cobalt content in tungsten carbide powder, securing the mechanical properties of the sintered WC-Co material such as hardness, compressive strength, and wear resistance [12]. Moreover, as the WC-Co substrate has a bonding property and coefficient of thermal expansion similar to those of the diamond coating layer, it is easier to generate nuclei for the deposition of a diamond coating film [5]. In this study, microcrystalline diamond (MCD) and nanocrystalline diamond (NCD) coatings with thickness of 8–10 μm were deposited on ball endmill tools by chemical vapor deposition. Coating was performed at the Korean branch of Oerlikon Balzers, and BALDIA COMPACT and BALDIA NANO coatings were applied to obtain the MCD and NCD-coated tools, respectively. 

**Figure 1:**
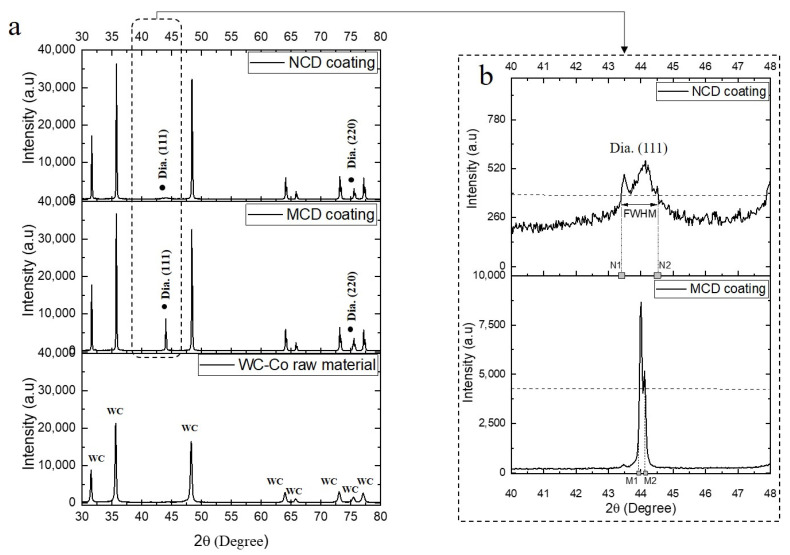
XRD patterns of (**a**) WC-Co substrate and MCD and NCD coating films. (**b**) Magnified view of crystalline plane (111). (FWHM, full width at half maximum).

The X-ray diffraction (XRD) patterns of the substrate and MCD and NCD coatings are shown in Figure 1a. Only a peak corresponding to tungsten carbide was observed for the substrate, while peaks at diffraction angles of 44° and 75.5°, which correspond to crystalline planes (111) and (222) of a cube diamond, respectively, were observed on the MCD and NCD coating surfaces [13]. At the diffraction angle corresponding to crystalline plane (111), for the MCD coating, the peak of the XRD pattern had a higher intensity than that of the NCD coating but a narrower width. For the NCD coating, the intensity was very weak compared with that of other peaks, but the peak around 44° was wider, as shown in Figure 1b. This is a typical difference between the MCD and NCD coatings. As shown in Figure 1b, the full width at half maximum of the NCD coating was larger than that of the conventional diamond coating [14]. This is due to the existence of nanocrystals and several grain boundaries in the coating layer. 

**Figure 2:**
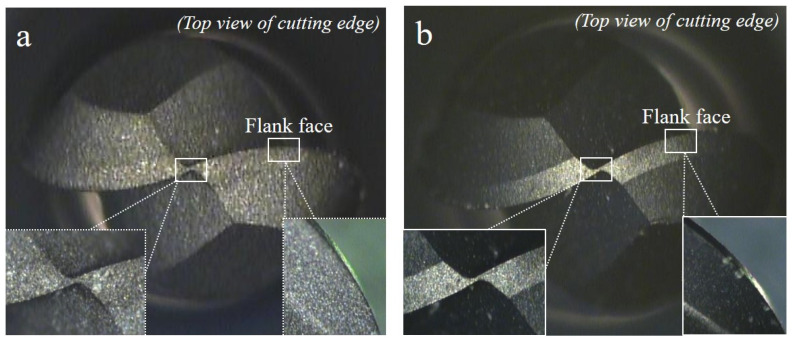
Optical microscope images of endmill tools with (**a**) MCD and (**b**) NCD coatings.

**Figure 3:**
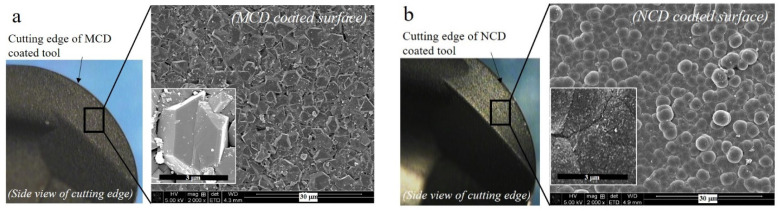
Morphology of (**a**) MCD and (**b**) NCD coatings.

Figure 2 shows optical images of the MCD and NCD-coated tools measured by magnifying an optical microscope by factors of 76 (main photographs) and 128 (insets). Figure 3 shows the coating surface morphologies observed by focused ion beam imaging. In the optical images in Figure 2, the grains of the MCD coating surface appear larger than those of the NCD coating surface, and thus, the NCD coating surface appears smoother. In the magnified images (insets in Figure 2), the NCD coating surface appears denser and the boundary line between the cutting edge and blade can be clearly observed. This is because the NCD coating surface is smoother owing to the grain size difference, and it better matches the dimensional tolerance of parts after coating compared with the MCD coating.

The crystal morphologies and sizes of the two coatings can be observed in the micro-morphology shown in Figure 3. In the MCD coating, cone-shaped crystals with a size of 3–5 μm form the surface, whereas in the NCD coating, nano-sized beads are assembled to form a morphology resembling a cauliflower. Unlike the optical microscope images, no notable difference is observed in roughness between the two coating surfaces in the morphologies obtained by focused ion beam–scanning electron microscopy. 

**Figure 4:**
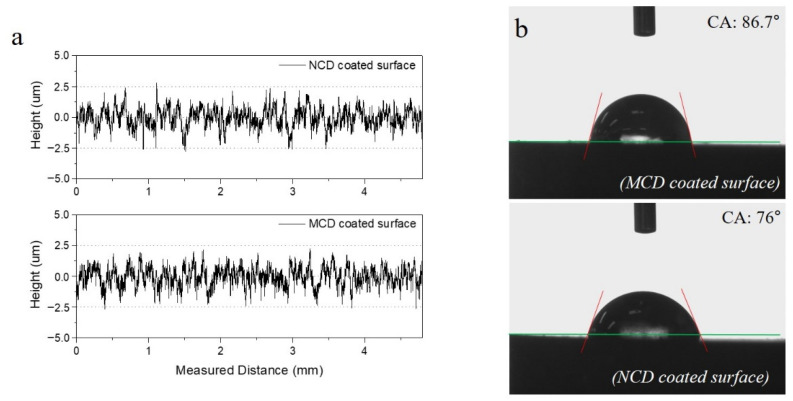
(**a**) Surface roughness and (**b**) contact angle of MCD and NCD-coated tools.

The measured surface roughness (Ra) was 0.618 μm for the MCD coating and 0.622 μm for the NCD coating, as shown in Figure 4a. The roughness of the NCD coating was slightly larger because the thickness and surface roughness of the nanocoating film changed in proportion [13,15]. Najar et al. [13] claimed that the thickness of a nanocoating film is typically 3 μm, and when the process variable is adjusted to increase the thickness, the surface roughness also increases. In this study, we deposited the NCD coating to obtain a thickness of 8–10 μm for comparison with the MCD coating. Similar to other studies, deposited grain boundaries were formed by upward agglomeration with grain boundaries aligned flat on the surface morphology, thereby decreasing the surface roughness. 

As shown in Figure 4b, the contact angle of the NCD coating was smaller than that of the MCD coating, as determined by a wettability evaluation of the two coating surfaces. The two coating surfaces had similar roughness, but the NCD coating, in which numerous nanobeads are gathered to form grain boundaries, had a relatively hydrophilic surface because its area was larger than that of the MCD coating. Depending on the wettability of the cutting tool surface, it can serve as a storage of lubricant in a lubrication environment to reduce the tool wear [16]. 

**Figure 5:**
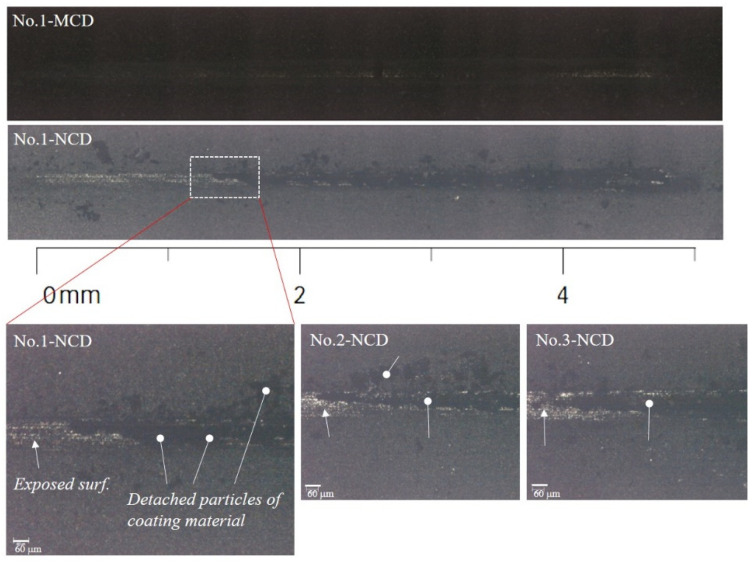
Micro-scratch test results.

The bonding strength between the substrate and coating is a parameter that determines the coating life and performance. This is because a strong bonding demands a large force to be exerted to the coating surface for generating delamination with the substrate [6]. The NCD coating is more prone to delamination and has a lower bonding strength than the MCD coating [17]. In this study, we performed micro-scratch tests while applying loads from 1 to 30 N and observed considerable delamination only in the NCD coating, as shown in Figure 5. From three tests, the critical loads at which delamination of the NCD coating occurred were 10.87, 13.3, and 17.27 N. The settings of the micro-scratch tests are listed in Table 4.

### 2.2. Measurement Equipment

The tool wear images before and after a high-speed milling were acquired using an optical microscope (ROI Instrument). The surface morphology of the MCD and NCD coatings was measured using a focused ion beam–scanning electron microscopy system (NOVA-600; FEI Company, Hillsboro, OR, USA). The roughness of the machined surface was measured using a surface roughness meter (SJ-410; Mitsubishi, Tokyo, Japan), and the roughness average was obtained by measuring at three points per machining method and step. The bonding strengths of the substrate and coating film were measured under a load of approximately 30 N using a micro-scratch tester. The spectrum of the coating layer by grain size was analysed using an XRD system (X’Pert-Pro MPD; Malvern Panalytical, Malvern, UK). The XRD profile was recorded using the CuKα radiation generated at 40 kV and 30 mA. Scanning was performed in the range of 2*θ* for 20–80° in intervals of 0.01° at a scan speed of 4°/min. 

### 2.3. Experimental Conditions and Procedures

**Figure 6:**
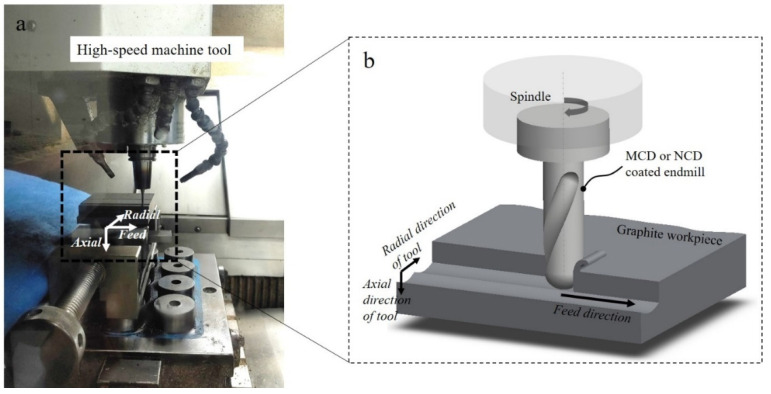
Experimental setup for high-speed machine tool: (**a**) photograph and (**b**) 3D model of machining area.

The machining experiment for the two coated tools was conducted using a three-axis CNC (computer numerical control) high-speed machine tool (RXP500; Röders TEC, Soltau, Germany). This tool has a maximum rotation speed of the principal axis of 42,000 rpm, maximum feed rate of 60,000 mm/min, and machining precision of ±2 μm. Figure 6 shows the setup of the milling test (Figure 6a) and the corresponding 3D model (Figure 6b). The graphite workpiece was fixed to the feed stage on the *XY* plane, and the diamond-coated ball endmill was mounted on the spindle. Precision machining was performed while removing the irregularities of scallop generated by rough machining. It was conducted in a dry environment, and the machining conditions were selected to comply with the recommended cutting ranges provided by the tool manufacturer, as summarised in Table 5.

**Figure 7:**
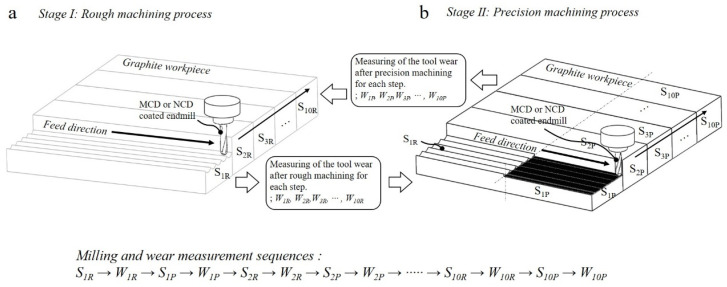
Sequence alternating milling processes of (**a**) rough and (**b**) precision machining.

**Figure 8:**
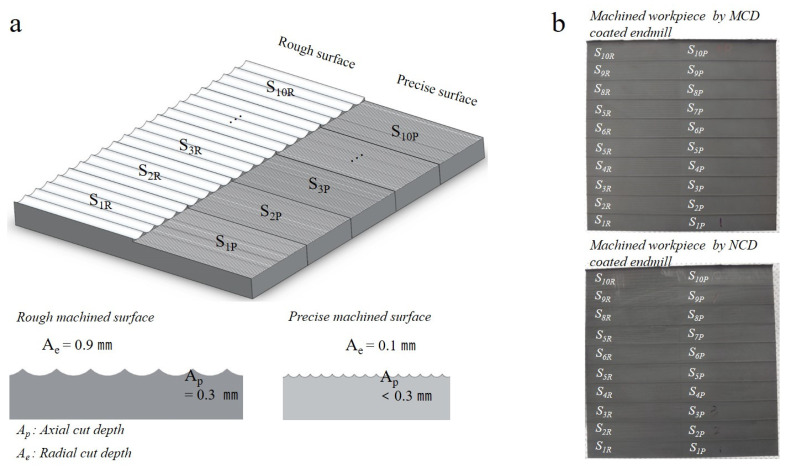
(**a**) Diagram of machined surface morphology after milling. (**b**) Optical microscope images of machined surfaces after rough and precision milling processes using MCD and NCD-coated tools.

High-speed milling was performed in the sequence shown in Figure 7 to compare the changes of the machined surface owing to wear and those generated during the rough and precision machining processes using the MCD and NCD-coated tools. Furthermore, the wear mode of coating, delamination time, and changes of machined surface (machinability) were closely observed over 10 steps for a total cutting length of 116.4 m (approximate cutting length per step of 11.6 m). After every step, the tool was unclamped, and the wear widths of the flank and rake faces were measured with a microscope. Then, the wear of the tool relative to the cutting length was compared and evaluated. Figure 8a illustrates the workpiece surface morphology after rough and precision machining. Figure 8b shows images of the workpieces machined using the MCD and NCD-coated tools. After the 10 steps, the machining performances of the two endmills were compared by measuring the surface roughness after rough and precision machining. The results were graphically represented according to the tool wear. The surface roughness was measured along the perpendicular to the tool feed direction and expressed as roughness average Ra. 

## 3. Results and Discussion

### 3.1. Tool Wear of Diamond-Coated Endmills

**Figure 9:**
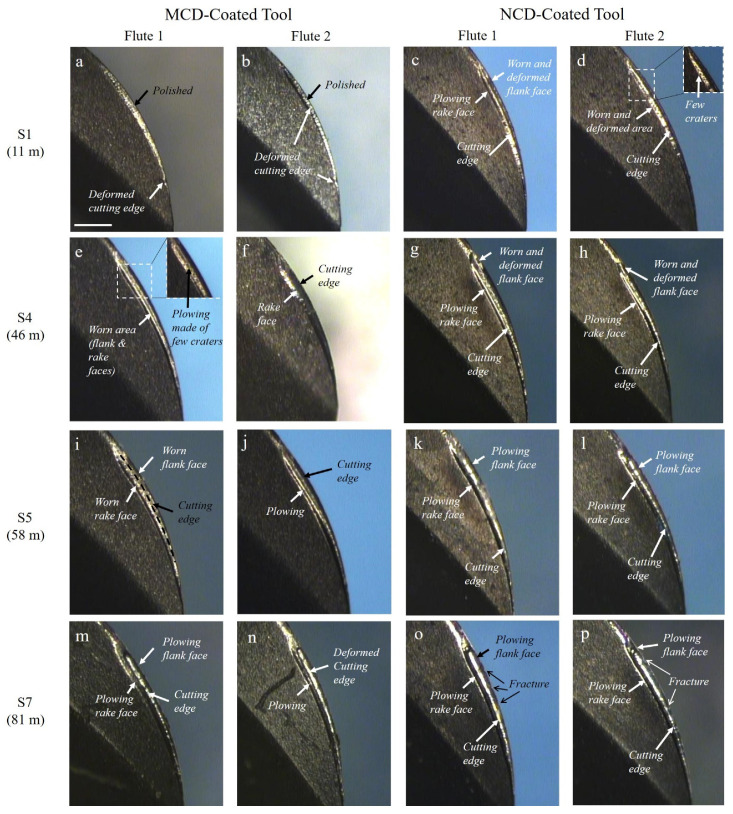

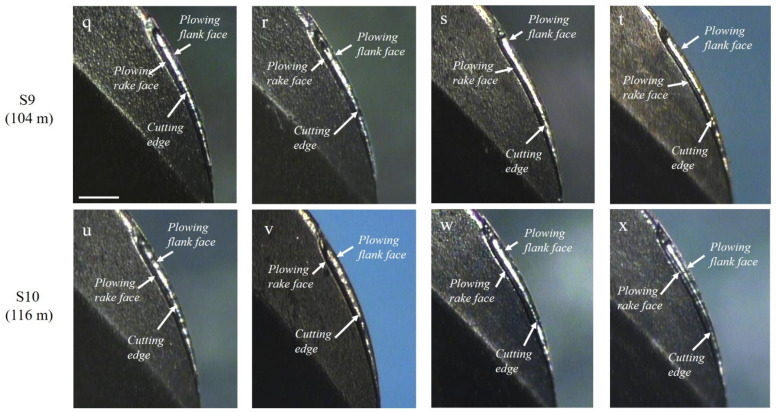
Side view of tool wear for MCD and NCD-coated tools during high-speed milling at steps (**a**–**d**) 1, (**e**–**h**) 4, (**i**–**l**) 5, (**m**–**p**) 7, (**q**–**t**) 9, and (**u**–**x**) 10.

**Figure 10:**
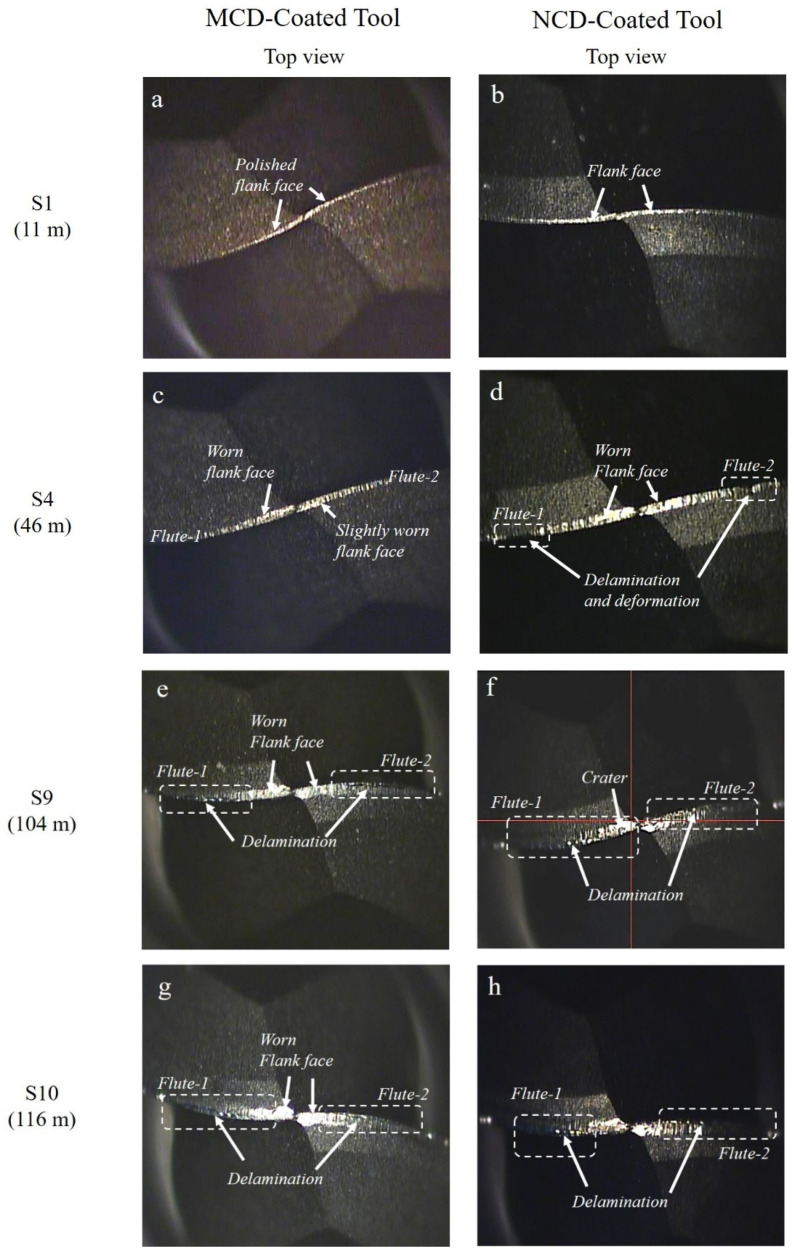
Top view of tool wear for MCD and NCD-coated tools during high-speed milling at steps (**a**,**b**) 1, (**c**,**d**) 4, (**e**,**f**) 9, and (**g**,**h**) 10.

**Figure 11:**
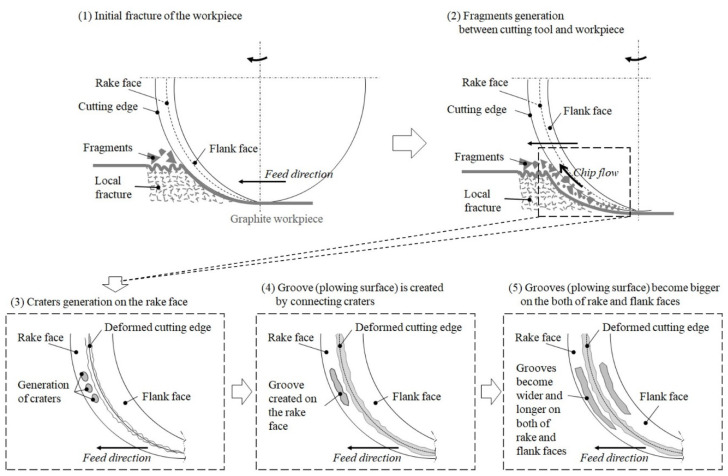
Wearing of flank and rake faces on diamond-coated tool.

The major wear trends of the MCD and NCD-coated ball endmills during high-speed milling are shown in Figure 9 and Figure 10. Regardless of the diamond grain size, the wear of the flank and rake faces of the tool relative to the cutting length progressed as shown in Figure 11: (1) polishing or grinding of the surface, (2) deformation of the cutting edge and crater wear of the cutting edge, (3) generation of ploughing-type lateral channel as the craters interconnect, (4) increase in lateral channel size of the flank and rake faces and in the deformation of the cutting edge, and (5) occurrence of the delamination of coating layer and substrate damage. Similarly, the wear of the MCD and NCD-coated tools showed the same trend, but the coating was largely delaminated at steps 9 and 4, respectively, corresponding to a large difference between coatings, as shown in Figure 10. Moreover, no notable damage was observed in the MCD-coated tool, but the NCD-coated tool showed damage of the cutting edge from step 7, as shown in Figure 9s,t.

Cabral et al. [4] explained the generation mechanism of a crater and lateral channel while turning graphite material using a diamond-coated tool. As the graphite powder moves from the cutting edge of the insert to the cutting edge of the side during machining, it causes coating delamination, generating craters and lateral channels. 

For continuous cutting (e.g., turning), machining is performed with the edge of the insert in continuous contact with the graphite material. Therefore, for graphite machining with the presence of fractured particles caused by local fractures on the surface, the particles can cause delamination of coating by flowing along the cutting edge at a high blasting speed from the top of the insert edge. 

For intermittent cutting (e.g., milling), Kanda et al. [18] found that the cracks in the diamond coating film are fractured as they propagate to the surface. The experimental results with various combinations of tool shapes, cutting processes, and workpieces showed that stress concentration at the coating interface causes cracks on the coating film, hindering the use of a diamond-coated tool. On the other hand, different patterns were found in [10,11]. Lei et al. [10] and Okada et al. [11] found that the dominant wear mechanisms of a diamond-coated tool were grinding, adhesion, and diffusion wear. In our study, craters and lateral channels similar to those appearing by turning were observed on the flank and rake faces around the cutting edge. 

By comparing our results with those from previous studies, the following wear similarities and differences between MCD and NCD coatings were observed. Regarding similarities, the size of diamond crystals did not act as a dominant factor in the activation of coating wear. In other words, the effects of repeated collisions with the graphite grains or a newly machined face on the diamond coating wear varied greatly by the machining conditions and tool size. These effects can be classified into two types. One type corresponds to micro-cutting, such as a micro-sized tool or machining conditions in the range of several micrometres. The graphite grains acting on the diamond coating surface damaged the coating layer by activating wear mechanisms such as grinding and adhesion. Another type is the damage of the coating layer by activation of the flat delamination wear mechanism, which corresponds to cutting under a general range of machining variables including high speed. According to Suh [19], the delamination wear generally occurs when the friction force generated by a sliding speed causes a plastic deformation on the subsurface of a relatively soft material. This deformation generates cracks around pre-existing void inclusions inside the material. Then, the voids and cracks interconnect, forming long cracks along the parallel to the surface. 

In this study, the crystalline structures of MCD or NCD coatings did not cause plastic deformation, which is a condition of delamination wear because the structures have a higher hardness than graphite and are brittle. However, the bonding strength on the coating interface is weaker than that between crystals, and the concentration of stress by external impact generates cracks on the subsurface (i.e., coating interface) at the same depth as the coating thickness. As a result, the wear behaviour is that of delamination wear [18]. Therefore, diamond coating applied to high-speed rough machining under general cutting conditions must have a large bonding strength with the substrate, and deposition should be stabilised to prevent voids or cracks at the bonding interface. Accordingly, the difference between the MCD and NCD coating can be explained along with the results of micro-scratch tests reported in Section 2.1. The delamination and damage of the cutting edge of the MCD coating film showed substantial differences from those of the NCD coating film because it had a much larger bonding strength with the substrate than the NCD coating. For MCD coating, delamination occurred after cutting for an additional length of 58 m, and the fracture of cutting edge was not observed until the end of machining. 

### 3.2. Relations between Machined Surface, Surface Roughness, and Tool Wear

**Figure 12:**
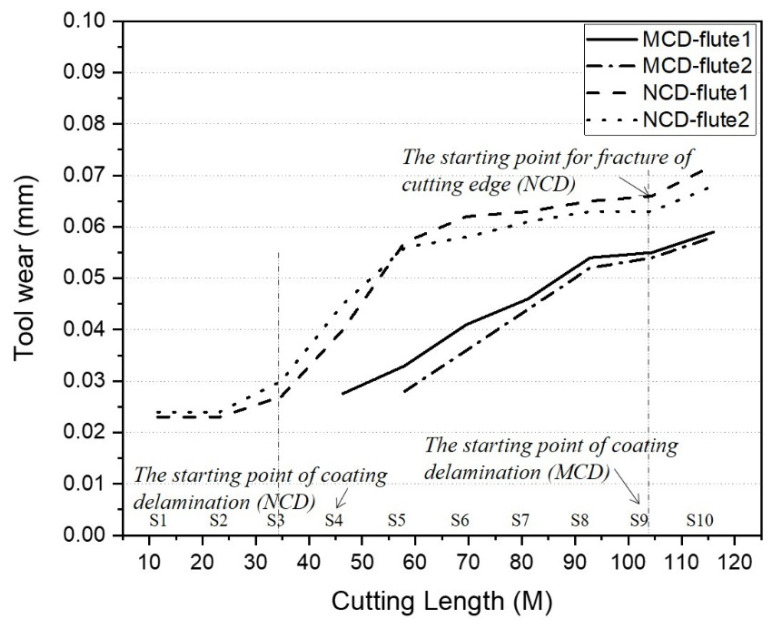
Comparison of tool wear according to cutting length of MCD and NCD-coated tools.

The wear widths of the tool flank and rake faces were measured in each step. The tool wear relative to the cutting length is shown in Figure 12. The NCD-coated tool showed a larger wear on both edges than the MCD-coated tool, and the difference tended to narrow as the cutting length increased. The total wear of the cutting edge of the MCD and NCD coating tools were 60 and 70 μm, respectively, and the service life of diamond-coated tools was determined by an overall wear of 100 μm [5]. In this study, we aimed to compare the wear change trends of MCD and NCD coating and the changes in the machining performance over wear steps. Thus, we terminated machining after coating delamination or damage in the MCD-coated tool, which had a much larger bonding strength. If coating is highly damaged and a large part of the substrate is exposed, the cutting edge undergoes rapid wear, and the machining performance decreases rapidly afterwards. 

Every material surface has a roughness value. When friction occurs by the contact of two surfaces, surface protrusions (i.e., surface roughness) contact first and polish the surface of the other material. After this *running-in* stage, the friction surface gradually wears, and the dominant wear mechanism can be identified [20]. Hence, we identified the occurrence of the wear types of crater, scar (groove), and delamination on the flank and rake faces to be the wear onset. In other words, we did not identify as wear the cases where only polishing of the coating surface or deformation of the cutting edge occurred. The wear amounts of flutes 1 and 2 of the MCD-coated tool in Figure 12 are indicated in steps 4 and 5, respectively. 

During high-speed milling, the wear of the NCD-coated tool increased and showing almost similar wear for both edges in every step. The wear of the tool progressed very slowly until the cutting length at step 3, and the wear progressed rapidly from step 3 to the cutting length at step 5. Then, the wear gradually increased until step 9, and the wear slope showed a tendency to increase from step 9 onwards. When we examine the wear shapes of the cutting edge, as shown in Figure 10d,f,h, a large delamination of coating occurred at step 4, and craters were observed at the end of the cutting edge at step 9. Therefore, the increasing slope of wear in a specific section was likely caused by delamination of the coating and damage to the cutting edge of the tool tip. This is a general trend in the friction/wear properties of coating materials. For instance, Lee et al. [21] evaluated the wear resistance of titanium alloy with a titanium oxide film on the surface and found that the coefficient of friction increased rapidly from the time of coating delamination and became close to the coefficient of friction of the base substrate. In general, when the friction coefficient of a material increases, its wear also increases, and a more severe surface damage (e.g., tearing, breakage) increases the friction coefficient curve fluctuation [22]. 

Wear according to the cutting length of the two cutting edges for the MCD-coated tool also showed a similar trend to that of the NCD-coated tool. The wear slope was lower than that of the NCD-coated tool, but the wear rapidly increased from steps 4 to 8. The same value was observed between steps 8 and 9, and the wear slightly increased between steps 9 and 10. The MCD-coated tool also showed a large delamination of the flank face in steps 9 and 10, and wear of the two edges started in steps 4 and 5. Thus, the two types of diamond-coated tools showed similar trends of wear change according to the cutting length, although the total wear showed a difference because the occurrence of delamination and damage changed according to the bonding strength of the coating layer. 

Wavy or clamshell-shaped scallops are generated depending on the type of tool applied to machined surfaces, and the width and height of these scallops are affected by the radial and axial cutting depths. In general, these cutting depths in rough machining are set several times larger than those in precision machining. Therefore, the scallops of precision machining have relatively narrow gaps and low height, whereas those of rough machining are larger. The scallops of a machined surface are important to determine quality because they are reflected on the machined surface along with the changed surface roughness owing to tool deformation during machining and damage by wear to the workpiece surface. For ball endmills, the area that participates in machining decreases according to the axial cutting depth, and approaching the tip of the cutting edge makes the rotation speed tend to zero, hindering normal cutting. 

**Figure 13:**
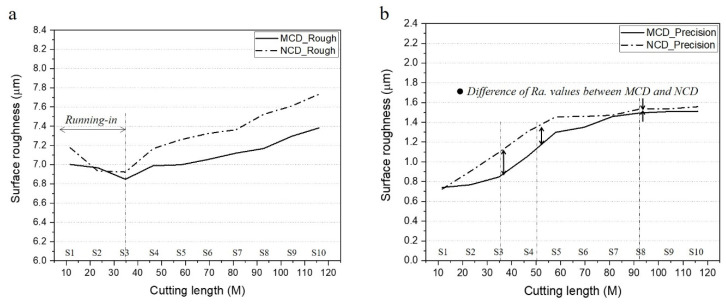
Roughness of machined surface according to cutting length for MCD and NCD-coated tools using (**a**) rough and (**b**) precision machining.

We considered that the change trend of surface roughness (i.e., machinability) in rough and precision machining can be completely different depending on the shape of the cutting-edge wear and deformation of the MCD and NCD-coated tools. Accordingly, we investigated the relations between various factors according to the change of cutting length. To this end, rough and precision machining processes were performed together in each step, and the corresponding surface roughness was measured. Regarding the machining process, as shown in Figure 7, rough machining was performed first, and then, only half of the machined area was processed using precision machining. Figure 13 shows the change trend of surface roughness according to the cutting length of the MCD and NCD-coated tools for rough and precision machining. The MCD and NCD-coated tools showed the following differences. First, in rough machining, as the cutting length increased, the difference in roughness between the two machined surfaces gradually widened. Second, precision machining showed the opposite trend, and surface roughness showed almost similar values from step 6. Third, except for the initial machining section (steps 1–3) of rough machining, both machining processes showed increasing surface roughness with the cutting length. 

**Figure 14:**
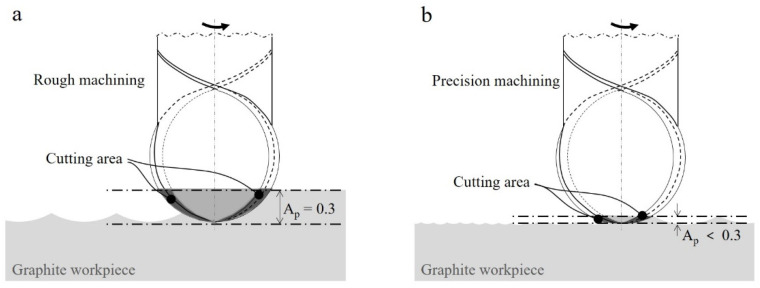
Area of tool edge participating in cutting during (**a**) rough and (**b**) precision machining.

The roughness of the machined surfaces with both tools decreased over time in the initial section of rough machining owing to taming, as discussed above. As the surface irregularities owing to coating undergo grinding in the beginning of machining, the tool surface roughness improves, thereby improving the roughness of the machined surface. Various studies on comparisons of diamond coating [5,14,17], including the one by Sun et al. [14], have reported that a smaller roughness average Ra of the coating surface decreases the roughness of the machined surface. Furthermore, as shown in Figure 14, the area of the coated edge participating in cutting during rough machining was larger than that in precision machining. Thus, the effect of the tool surface roughness on the machined surface may be larger in rough machining than in precision machining. For this reason, roughness improving of the machined surface was observed early during rough machining but not during early precision machining.

As a clear difference in the roughness change between the surfaces machined by the two tools, during rough machining, the difference in surface roughness relative to the cutting edge increased, whereas the opposite trend was observed during precision machining. We set the axial cut depth to 0.1 mm during precision machining, which is very small compared with the diameter of the ball endmill of 3.0 mm. Therefore, only a small area of the ball tip performed cutting according to the tool rotation axis where the two cutting edges intersected. For micro-precision cutting, coating and workpiece surface damage occurred owing to the deformation or chips of the cutting edge. Thus, the cutting-edge radius resulting from the coating became important to determine the quality of the machined surface. To identify dominant factors that caused the change of the machined surface roughness during precision machining, the machined surface damage caused by difference in the coated tool radius and diamond or graphite fragments was analysed. In this study, cutting edge deformation of the two coated tools owing to machining was not clearly observed. Therefore, deformation was excluded from this analysis.

**Figure 15:**
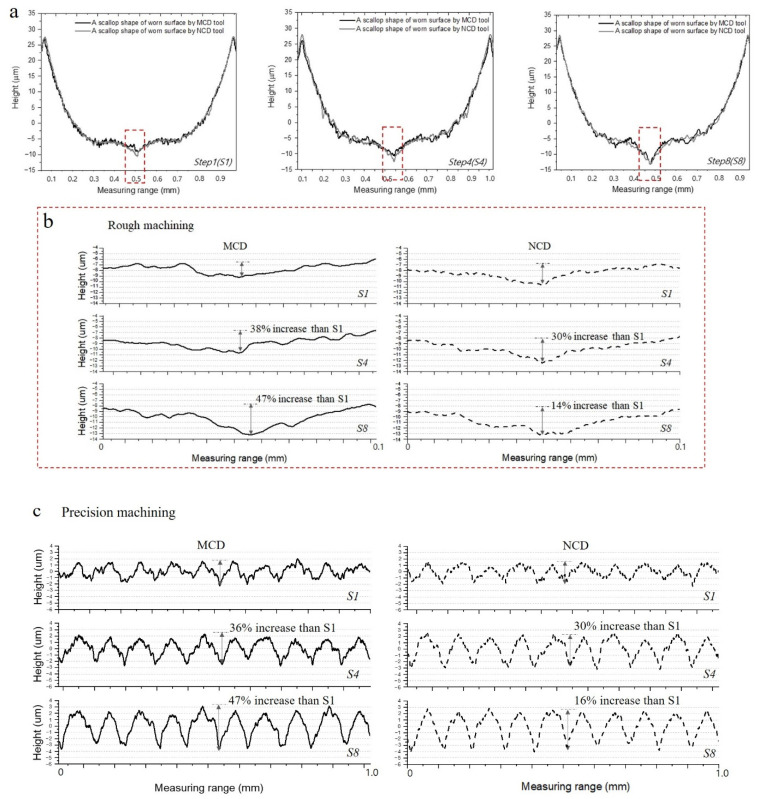
Surface profiles machined using MCD and NCD-coated tools. (**a**) Magnified graphs of scallops on machined surface during rough machining, (**b**) magnified profile of the groove within the red box area in (**a**), and (**c**) magnified graphs of machined surface during precision machining.

Yu and Cheong [23] reported that a smaller cutting-edge radius allows shorter micro-cutting because the radius and radial cut depth directly affect the size of scallops generated on the surface. The MCD and NCD-coated tools used in this study clearly showed differences in the curvature of the cutting edge and the rake face edge, as shown in Figure 3. If a change in the cutting-edge radius influenced precision machining using the two tools, there must be a difference in the machined surface scallops during early cutting, when coating wear is the smallest. This is because a scallop reflects the shape of the cutting edge. Therefore, the scallops of machined surfaces during rough and precision machining using the NCD-coated tool, which has a sharper cutting edge, must be smaller than those using the MCD-coated tool. However, such a trend was not observed on the machined surfaces during rough and precision machining in this study. The corresponding analysis results are shown in Figure 15.

**Figure 16:**
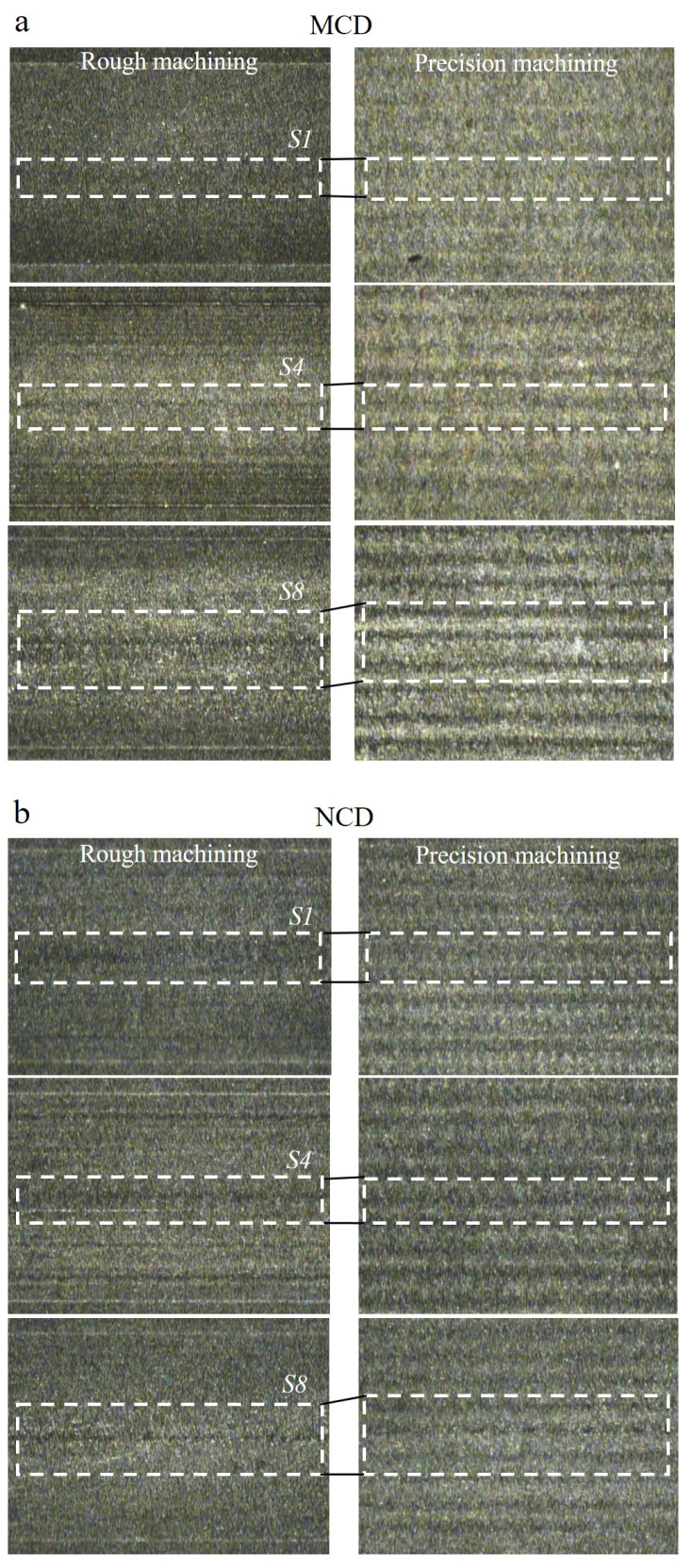
Machined surfaces compared to cutting length using (**a**) MCD and (**b**) NCD-coated tools. (S1, S4 and S8—Each section of cutting length; White box—The contact area between the center of the cutting edge and the machining surface).

The coating grains and graphite fragments that came off changed the profile of the machined surface scallop and finally changed the surface roughness during precision cutting. The abrasive wear is often considered as a type of cutting because the generation of the valley (groove), a representative abrasive wear, is similar to cutting process [24]. The profile shown in Figure 15a indicates an inaccurate hemisphere shape of the cross-section of the ball endmill. The section of approximately 0.25 mm around the rotation axis of the tool showed a valley shape, and the groove became deeper as the cutting length increased. The rough machined surface was re-machined during precision cutting. Thus, the change rate of the valley depth according to the cutting length was analysed after enlarging the length (0.1 mm) around the rotation axis of the scallop of the machined surface during rough machining (as shown in Figure 15b). The change rates of the valley depth compared with cutting length S1 were 38% and 47%, respectively, for the MCD-coated tool. Thus, they increased as the cutting length increased. In contrast, the change rates of the NCD-coated tool were 30% and 14%, showing a decreasing trend as the cutting length increased. This trend was the same with the surface profile for the 1.0 mm section after precision machining, as shown in Figure 15c. This may be because precision cutting was performed with the concept of grinding to the large irregularities on the surface caused by rough machining. If large irregularities with 0.9 mm gaps are machined with only a radial cut depth of 0.1 mm without axial cut depth, damage which generated during pre-processing, may be remained on the precision machined surface without being fully removed. Such damage is particularly related to the contact between the surface and tool centre. This can be seen in Figure 16, which shows images of the surfaces machined by the two tools. The similar surface roughness obtained from precision machining using the two coated tools as the increasing cutting length can be explained as follows. As the NCD coating wears faster, the initial wear of the machined surface caused by NCD grains is larger than that observed with the MCD-coated tool. However, as the wear of the MCD coating with larger fragments continued, it showed a larger grinding wear rate and thus degradation of the surface roughness during precision cutting. 

## 4. Conclusions

We conducted high-speed milling tests for rough and precision machining on graphite (G5) workpieces intended for glass moulding, considering that G5 graphite is a material with high hardness and brittleness. In addition, changes in wear according to the cutting length and changes in the machined surface roughness using MCD and NCD-coated tools were analysed in detail. The following conclusions were drawn assuming the same initial surface roughness when using the two coated tools:

1. The wear of the tool flank and rake faces progressed the same during machining regardless of the type of crystalline diamond coating. However, the total tool wear differed as the delamination and damage times changed according to the adhesion strength of the coating layer.
2. The size of the diamond crystal was not a dominant factor on coating wear, but it had a major impact on the wear of the machined surface in contact with the cutting edge centre during precision machining. Therefore, despite an increasing cutting length, the NCD-coated tool with a fine grain size showed a decrease in the abrasive wear rate of the machined surface.
3. For rough machining, delamination wear was the wear mechanism for the MCD and NCD coatings. Therefore, the MCD coating, which had a higher bonding force with the substrate, showed a higher performance in terms of service life and machinability.
4. When rough and precision machining processes were performed with one tool, the two coated tools provided similar roughness on the final machined surface.

We found through high-speed milling tests that the bonding strength and grain size of the coating had significant effects on rough and precision machining by analysing the relations between wear according to the cutting length and machinability of MCD and NCD-coated tools. Coatings can improve the service life and machinability of tools for hard-to-machine materials. Nevertheless, it has weaknesses such as low quality of the machined surface and acceleration of tool wear owing to hard fragments discharged after delamination onset. 

Overall, for rough machining with a large contact area between the cutting tool and workpiece, the bonding strength of the coating and the defects inside the coating act earlier than the grain size of the coating, affecting the quality of the machined surface. For precision machining over a micro-area of the cutting tool, the grain size of the coating has a major impact on the abrasive wear control of the machined surface, and a diamond-coated tool with a small grain size is more advantageous for obtaining precise surface machining. The findings of our study along with those of previous studies may provide guidelines for selecting tools to perform suitable rough and precision machining of hard graphite materials using diamond-coated tools.

## Figures and Tables

**Table 1:** Mechanical properties of G5 workpiece. (Refer to the datasheet of G5 provided by Mersen).

Mechanical Property	Value
Bulk density (g/cm^3^)	1.84
Grain size (μm)	7
Flexural strength (MPa)	72
Hardness Rockwell “H”	90

**Table 2:** Tool specifications.

Geometry	Parameter	Value
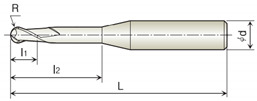	Radius *R* (mm)	1.5
	Diameter of tool shank *d* (mm)	6.0
	Cut length *l*_1_ (mm)	8.0
	Effective length *l*_2_ (mm)	16.0
	Tool length *L* (mm)	60.0
	Helix angle *θ* (°)	30
	Flute number *z*	2

**Table 3:** Characteristics of WC-Co raw material (Grade: GK05A).

Grain Size (um)	Cobalt (%)	Density (g/cm^3^)	Hardness (HV_30_)
1.0	6.0	14.9 ± 0.1	1740 ± 50

**Table 4:** Parameters of micro-scratch tests.

Scratch Parameter	Value
Load range (N)	1–30
Load rate (N/min)	58
Acoustic emission sensitivity	9
Scanning load (N)	0.1
Speed (mm/min)	10
Length (mm)	5

**Table 5:** High-speed milling parameters.

Milling Parameter	Value
Cutting velocity *V*_c_ (m/min)	104
Spindle speed *N* (rpm)	11,000
Feed rate *F* (mm/min)	1200
Feed per tooth *f*_z_ (mm/tooth)	0.055
Axial cut depth *A*_p_ (mm)	0.3 (rough machining)/<0.3 (precision machining)
Radial cut depth *A*_e_ (mm)	0.9 (rough machining)/0.1 (precision machining)
Total cutting length (m)	104.4 (rough machining)/12 (precision machining)
Lubrication environment	Dry

## Data Availability

Not applicable.
